# Editorial: Understanding IGF-I's impact on neurocognitive aging: insights and therapeutic directions

**DOI:** 10.3389/fnagi.2026.1973617

**Published:** 2026-09-11

**Authors:** Jonathan Adrián Zegarra-Valdivia, Ignacio Torres-Aleman, Angel Nuñez

**Affiliations:** 1 Achucarro Basque Center for Neuroscience, Leioa, Spain; 2 Universidad Científica del Sur, Lima, Peru; 3 Department of Anatomy, Histology and Neuroscience, Faculty of Medicine, Universidad Autónoma de Madrid, Madrid, Spain

**Keywords:** IGF-I receptor (IGF-IR), insulin-like growth factor I (IGF-I), neurocognitive aging, neurodegeneration, neuroprotection

Aging influences the IGF-I system in many ways, including changes in circulating IGF-I levels, its access to the brain, local production, receptor levels, intracellular signaling, and its effects within specific neuronal, glial, and vascular areas. Therefore, the biological effects of IGF-I cannot be understood solely based on its circulating or tissue concentrations. Rather, current evidence supports a context-dependent model in which IGF-I acts as an integrative signal coordinating cellular maintenance, synaptic and circuit function, metabolism, sleep–wake regulation, mood, and cognition (Nuñez et al., 2023; García-Magro et al., 2025; Zegarra-Valdivia et al., 2025a). The contributions to this Research Topic collectively support this view, highlighting the importance of considering regional, cell-type-specific, temporal, and disease-related differences in IGF-I signaling to better understand its role in neurocognitive aging.

Chen et al., in “*Insulin-like growth factor 1 associated research in Alzheimer's disease: an exploratory trends analysis*,” describe how the Alzheimer's disease (AD) literature has expanded from early work on amyloid-β, tau, apoptosis, and neuroprotection toward insulin/IGF resistance, oxidative stress, cognition, depression, biomarkers, and translational applications. As a bibliometric mapping study, it cannot determine which mechanisms are causally involved or may represent viable therapeutic targets. Rather, its value lies in illustrating the field's progressive expansion and highlighting the persistent gap between a substantial body of preclinical evidence and its still-limited clinical translation (Chen et al.). This gap is particularly relevant given that systemic IGF-1 levels may not accurately reflect IGF-1 signaling in the brain, and the effects of modulating this pathway may vary by disease stage, brain region, and cellular target.

Yue et al., in “*Cell-type-dependent decline of IGF-1 and IGF-1R in the prefrontal cortex contributes to age-related behavioral changes*,” provide a detailed analysis of age-related changes in the IGF-1 system at both the regional and cell-type-specific levels. In the mouse prefrontal cortex, IGF-1 declined in both CaMKII-positive excitatory neurons and GAD67-positive inhibitory neurons from middle age onward, whereas IGF-1R declined selectively and progressively in excitatory neurons. Conditional reduction of *Igf1r* in prefrontal pyramidal neurons of young adult mice was sufficient to induce anxiety-like behavior across three behavioral paradigms, along with reduced social interaction and motivation, and impaired novel-object discrimination (Yue et al.). It is worth noting that these behavioral alterations occurred in the absence of detectable changes in open-field locomotor activity. These findings indicate that impaired IGF-1R signaling in prefrontal excitatory neurons is sufficient to reproduce specific behavioral features associated with aging. However, they do not establish that reduced IGF-1R signaling in these neurons is necessary for the behavioral changes that occur during natural aging, and the cognitive effects identified in this study are limited to recognition memory. Thus, the study highlights the value of cell-type-specific approaches for dissecting the contribution of IGF-1 signaling to age-related behavioral changes, while also emphasizing the limitations of extrapolating these findings to the broader neurocognitive aging process.

Champarini et al., in “*Intracisternal IGF-1 gene delivery attenuates early anxiety-like behavior but not dopaminergic neurodegeneration in a 6-OHDA rat model of parkinsonism*,” extend this distinction to a neurodegenerative context. Anxiety-like alterations emerged before overt motor impairment in the 6-OHDA model, and post-lesion intracisternal IGF-1 gene delivery attenuated these behavioral changes. However, the intervention did not preserve tyrosine hydroxylase-positive neurons in the ventral tegmental area. This dissociation between behavioral improvement and dopaminergic neuroprotection has mechanistic significance, implying that IGF-1 might modulate the functions of affected neural circuits or activate compensatory processes without outright preventing the neurodegenerative disease itself (Champarini et al.). These results underscore the need to differentiate between symptomatic relief, functional compensation, and genuine disease-modifying effects when assessing IGF-1-related treatments.

Yin et al., in “*Serum proteomics reveals biomarkers for diagnosis, stratification, and mechanistic insights into cerebral microbleeds*,” broaden the Topic toward neurovascular aging. Their serum proteomic analysis identified candidate biomarker panels for diagnosis and subtype discrimination and revealed distinct patterns of pathway enrichment by cerebral microbleed location, including IGF-related signaling in lobar microbleeds. These findings suggest a potential link between IGF signaling, cerebral small-vessel disease, extracellular matrix remodeling, and amyloid-associated vascular pathology (Yin et al.). However, interpretation is limited by the cross-sectional, single-center design, the relatively small sample size, the exclusion of mixed microbleeds, the absence of external validation, and differences in vascular characteristics across clinical groups. Accordingly, the association with IGF-related signaling should be regarded as hypothesis-generating rather than as evidence for a causal role of the IGF pathway or its potential therapeutic modulation.

Taken together, these studies are consistent with a broader body of evidence indicating that brain-derived IGF-I contributes to hippocampal long-term potentiation and spatial memory, that local IGF-I administration can restore impaired cortical response facilitation in aged mice, and that astrocytic IGF-IR may protect against ischemic damage and blood–brain barrier disruption (Herrero-Labrador et al., 2023; García-Magro et al., 2022; Suda et al., 2024). Collectively, these findings also support the view that IGF-I functions not only as a trophic factor but also as a dynamic modulator of brain activity and functional states (Nuñez et al., 2023; Zegarra-Valdivia et al., 2025b). Moreover, lifestyle-related conditions can modify brain responsiveness to circulating IGF-I without corresponding changes in serum levels, underscoring the limitations of using peripheral IGF-I concentrations as a surrogate marker of IGF-I activity within the brain (Zegarra-Valdivia et al., 2025a).

This synthesis suggests several directions for future translational research. IGF-I should be viewed as a functional signaling system rather than evaluated solely by ligand levels; it should also include measures of receptor expression, target engagement, downstream signaling, and circuit-level function. Similarly, intervention studies need to consider factors like the brain region, cell population, disease stage, sex, route of administration, dose, and therapeutic window. It is also crucial to distinguish IGF-I's effects on behavioral or cognitive improvements from neuroprotection or disease modification. Combining various techniques—such as proteomics, imaging, electrophysiology, and neuropsychological assessments—may help identify distinct cellular subgroups and clarify whether changes in the IGF-I system indicate impaired signaling, compensatory adaptation, or decreased responsiveness.

The main message of this Research Topic is that IGF-I activity should not just be increased or decreased. Instead, effective translation likely requires restoring or adjusting IGF-I signaling within the appropriate cellular, circuit, and temporal context. This precision-focused approach could offer a more practical way to understand vulnerability and resilience in brain aging and to create interventions targeting its cognitive, emotional, motor, and vascular aspects.
